# Bacterial Contamination of Drinking Water in Guadalajara, Mexico

**DOI:** 10.3390/ijerph16010067

**Published:** 2018-12-27

**Authors:** Francesca Rubino, Yahaira Corona, José Guadalupe Jiménez Pérez, Charlotte Smith

**Affiliations:** 1 Division of Environmental Health Sciences, School of Public Health, University of California, Berkeley, CA 94720, USA; rubinofrancesca@berkeley.edu; 2 Instituto de Investigaciones Tecnológicas del Agua, Zapopan, Jalisco 45088, Mexico; yahaira.corona@iitaac.org.mx (Y.C.); jose.jimenez@iitaac.org.mx (J.G.J.P.)

**Keywords:** Guadalajara, coliform, intermittent water supply, Colilert, tanks

## Abstract

In many regions where drinking water supply is intermittent and unreliable, households adapt by storing water in cisterns or rooftop tanks. Both intermittent supply and stored water can be vulnerable to contamination by microorganisms with deleterious health effects. The Metropolitan Zone of Guadalajara is a rapidly growing urban center with over five million residents where household storage is nearly ubiquitous. This pilot study was conducted in July 2018 to examine the microbiological quality of drinking water in Guadalajara. Samples were tested for free available chlorine residual, total coliform bacteria, and *Escherichia coli*. A survey on access to water and public perspectives was also conducted. Water exiting rooftop tanks exceeded regulatory limits for total coliform levels in half of the homes studied. Piped water arriving at two homes had total coliform levels that far exceeded regulatory limits. No *E. coli* were detected in any of the samples. Only 35% of homes had a chlorine residual between the recommended 0.2 and 1.5 mg/L. Many homes reported unpleasant odors and colors. Only 7% of residents drank the piped water. Future studies are needed, especially during April and May when many homes reported a higher disruption to water service.

## 1. Introduction

A key target of the Millennium Development Goals was to halve the proportion of the population without access to safe drinking water by 2015. In an effort to increase access, piped water supply systems have quickly expanded in developing countries such as Mexico. Between 1990 and 2015 the percentage of Mexico’s population with access to “improved” drinking water increased from 82% to 96% [1]. “Improved” denotes the construction of the water system (e.g., piped water, protected well). However, studies suggest that these “improved” drinking water systems are not analogous to clean, safe drinking water systems when assessed instead by water quality criteria [2,3,4].

A major issue with “improved” drinking water systems in developing nations is the lack of consistent water supply. Previous studies have shown that the changes in hydraulic pressure due to intermittent water supply (IWS) lead to microbial contamination that can cause dangerous gastrointestinal illnesses [5,6,7,8,9]. For example, Kumpel et al. found that samples from taps in Hubli-Dharwad, India, supplied intermittently were more likely to be contaminated by both total coliforms and *Escherichia coli* than taps supplied continuously [5]. Similarly, Erickson et al. demonstrated in Panama that IWS created hydraulic conditions which increased risk for contamination. Despite these impacts, water quality generally met relevant standards [8]. In addition to these changes in hydraulic pressure due to IWS, household practices to cope with IWS can introduce microbial contamination at the point of use [10]. Many homes in Mexico store water either in rooftop tanks or in underground cisterns in order to maintain access to water when there is a lapse in the supply. This practice may affect the risk of diarrheal disease [11,12].

**Figure 1:**
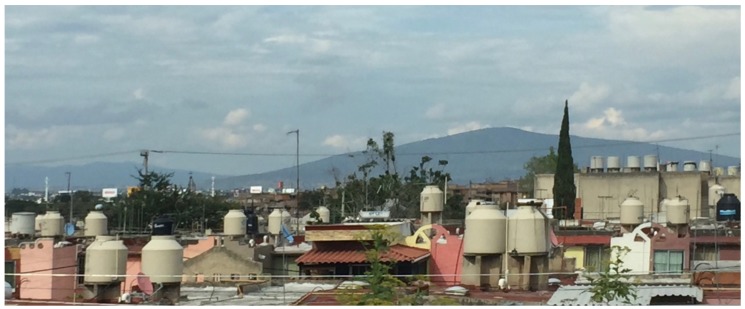
Typical rooftop tanks in Guadalajara.

This study focused on Mexico’s second largest city, where the practice of using cisterns and rooftop tanks is nearly ubiquitous (Figure 1). The Metropolitan Zone of Guadalajara (ZMG) encompasses the municipalities of San Pedro Tlaquepaque, Tonalá, Zapopan, Tlajomulco de Zúñiga, El Salto, and Guadalajara. The region is the second most heavily populated in Mexico, with a population that surpassed five million in 2017 [13], and is expected to reach seven million by 2025 [14]. Rapid population growth and over-exploitation of the water supply have resulted in a severe water crisis [15]. The primary water source, Lake Chapala, provides around 60% of the region’s water [16]. Wells and the Río Calderón serve as secondary sources. Water in the ZMG is provided by the Sistema Intermunicipal de los Servicios de Agua Potable y Alcantarillado (SIAPA). Despite high public concern about the quality of drinking water from these sources, access to independent studies on water quality and data from SIAPA have been limited. The objective of this study was to describe possible issues connected with IWS in the ZMG through a two-fold approach. The first part of the study analyzed biological, chemical and physical parameters linked to microbial contamination. The second part collected information on the public’s perception of water quality.

## 2. Materials and Methods

**Figure 2:**
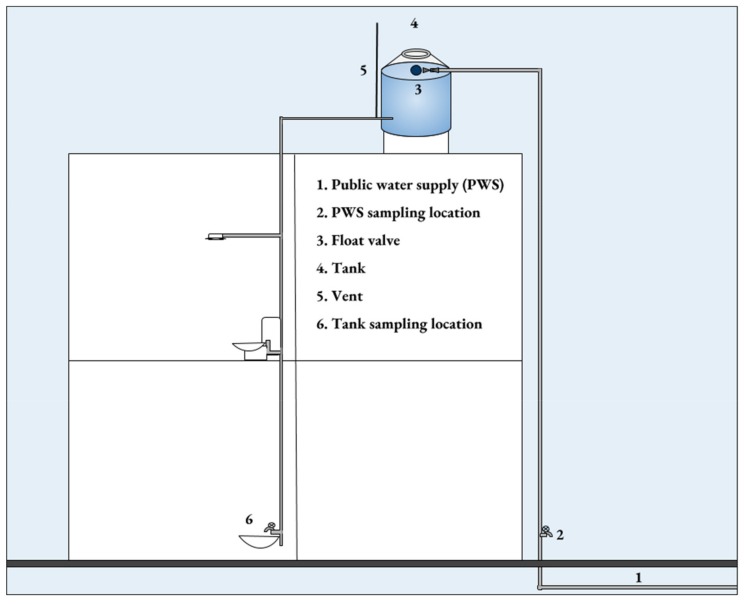
Typical ZMG plumbing infrastructure.

Sampling took place in July 2018 during the rainy season. Publicly available maps did not fully delineate the source of water in regions where water service has recently been added. Therefore, an AquaPro™ AP-2 conductivity meter (HM digital, Redondo Beach, CA, USA) was used at each house to determine the water source (Standard Methods No. 2510 [17]). As conductivity of the three water sources (Lake Chapala, wells, and Río Calderón) is quite different, we used that parameter to differentiate the sources. We determined the conductivity in water from houses where the source was known, then used those values to determine the source where it was unknown. Residential housing stock located on public streets in the ZMG supplied by SIAPA is highly uniform. Therefore, homes used in the study are typical of homes in the ZMG. Figure 2 illustrates the typical water supply infrastructure in the region. Rooftop tanks are automatically filled from the public water supply with system pressure via a float valve. Samples were taken from a tap supplied directly from the mains (number 2 in Figure 2) and from a tap supplied directly from the storage tank (number 6 in Figure 2). Initial chlorine residual was measured at 51 houses. Samples were taken for coliform bacteria and *E. coli* at 10 of the 51 houses. At these 10 houses, a repeat chlorine residual sample was taken 120 h after the initial sample. A map was created to illustrate the chlorine residual values using QGIS.

To limit ancillary variables, samples taken for bacteriological testing were from areas within the ZMG serviced by the main water provider, SIAPA, and supplied from the main water source, Lake Chapala. The ten homes were chosen based upon willingness to participate, dependency on a rooftop tank, and proximity to the other homes such that all ten homes were accessible by vehicle within a six-hour round-trip. This six-hour maximum holding time between collection and incubation is recommended by the World Health Organization [18]. Within each neighborhood, houses were approached until a person agreed to participate, or a local contact was able to facilitate access. Each home was tested twice at the same time of day. Samples were collected in 125 mL sterile screw-top polystyrene bottles containing sodium thiosulfate. Samples were transported on ice according to Standard Methods [17]. The Hach DR900™ (method 8021) was used to test for residual chlorine, the IDEXX Colilert-18™ Most Probable Number (MPN) test was used to test for total coliform bacteria, and the IDEXX Colilert-18™ test for fluorescence was used to test for *E. coli*. Analysis of results were compared to the standards stated in the revised Official Mexican Standard NOM-127-SSA1-1994 [19].

Prior studies have recognized the numerous factors that make testing for coliform bacteria in low-resourced settings challenging [20,21,22]. One particular challenge is meeting standards on controlled temperatures during transport and incubation. No incubators were available, nor were they obtainable in the region for the duration of the project and within the budgetary constraints. Due to these limitations, an incubator was constructed from a reptile heating lamp, a Styrofoam container, and aluminum foil. A cloth sheet was placed between the lamp and the container to prevent bright light from reaching the samples. The makeshift laboratory and incubator were sterilized with alcohol. The incubator’s ability to maintain a temperature of 35 °C was evaluated 24 h prior to sample incubation. During sample incubation, the temperature of the water (in vials containing distilled water that were placed in the center and the periphery of the incubator) was measured every 3 h over 28 h. Recorded temperatures ranged from 33.5 °C to 36.5 °C. Bacteriologic sample results were analyzed after 24 h and again after 28 h.

In addition to the water quality tests, a brief survey on water access and perception was conducted using Survey123™ (ESRI, Redlands, CA, USA), a mobile-GIS application. Permission for sampling was obtained and the survey was conducted by the local non-profit organization Instituto de Investigaciones Tecnológicas del Agua (IITAAC) in Spanish. They conducted the survey during sample collection. Homeowners’ verbal consent was obtained to collect samples after explaining the purpose of the study and that no one was obliged to answer any questions. No personal identifiers were obtained. Participants were asked about their water storage devices, how often they cleaned them, how they used the piped water, the intermittency of the supply, and the perceived aesthetic quality of the water. A total of 61 surveys were completed; the chlorine residual samples were taken from 51 of these homes.

## 3. Results

### 3.1. Survey

A summary of the survey results is shown in Table 1 and Table 2. Only four families out of the 61 surveyed consumed water from the public supply. One of these families filtered the water before consumption. Respondents indicated that they purchase bottled water for drinking. We did not assess perceptions of purchased water in terms of satisfaction with water quality or trust in its purity. Most households (95%) used the water for cleaning fruits and vegetables. However, it was common practice to filter or add a disinfectant when washing fruit and vegetables. Respondents indicated that they use “Microdyn” and other commercially available biocides to wash fruit and vegetables. An assessment of the efficacy of these practices was beyond the scope of this study. Only 20% of households used the water for cooking. Again, it was common practice to treat the water before use by boiling or filtration. Nearly 50% of the homes reported unpleasant odors and 59% reported unpleasant colors. During sampling, water supplied from the mains was noted to be brown or yellow at multiple homes, presumably as a by-product of corrosion in the iron mains. The corrosion by-products apparently settle in the tanks because water from the tanks in the same home was not discolored. Odors of high chlorine levels or hydrogen sulfide were observed at several locations. Interviewees reported a higher disruption to water service and increased odors and colors in spring, particularly in April and May. Public notification during new main installation, main repair or flushing when water is likely to experience temporary changes in quality is considered “best practice”. However, although not part of the survey, respondents explained to the interviewer that they rarely receive notice of infrastructure work that would affect their water quality and that dirty water does not seem to be related to any construction or maintenance activities.

The survey showed that most households depended on rooftop tanks, and that rooftop tanks were more common than cisterns. Additionally, at least one household was not actively using the house’s cistern. Asbestos rooftop tanks were more common in older regions of the city. Only six houses that were visited had rooftop tanks made with asbestos. The other 45 rooftop tanks were plastic Rotoplas™ models. Of the 34 homes with cisterns, 11 were asbestos. The other 22 were plastic or concrete. Over 42% of people surveyed had not cleaned their water storage devices within a year of the study. The longest anyone remembered cleaning their tanks was 10 years prior to the study. Of the 51 households with tanks, eight had never cleaned their tanks. One person rented their home and did not know when or if the tank was cleaned.

### 3.2. Chlorine Residuals

**Figure 3:**
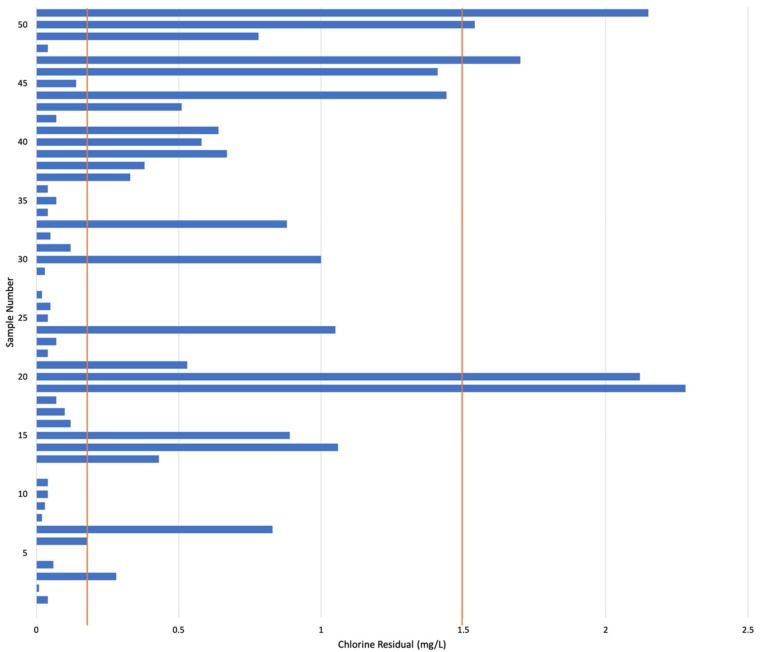
Chlorine residuals of 51 samples in the ZMG, July 2018. Mexican drinking water standards require chlorine residual to be between 0.2 and 1.5 mg/L; this range is demarcated by the red lines.

**Figure 4:**
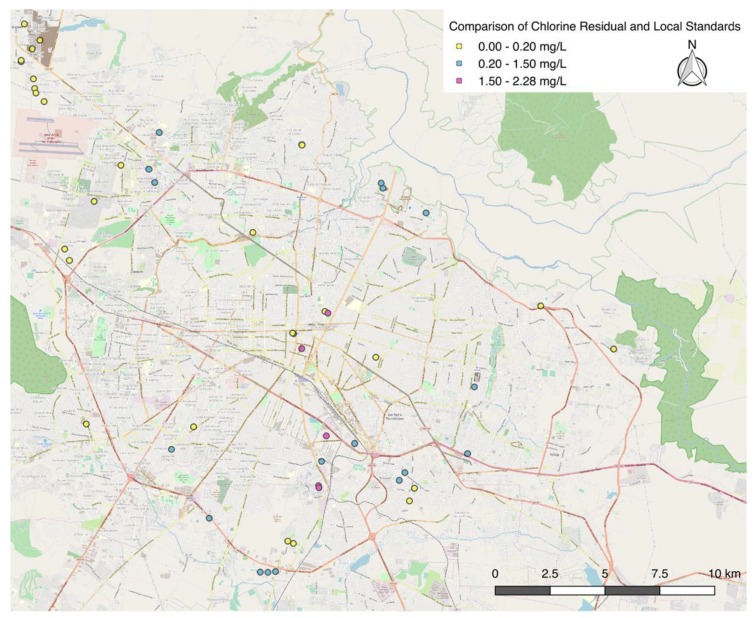
Map of chlorine residuals in the ZMG, July 2018.

**Figure 5:**
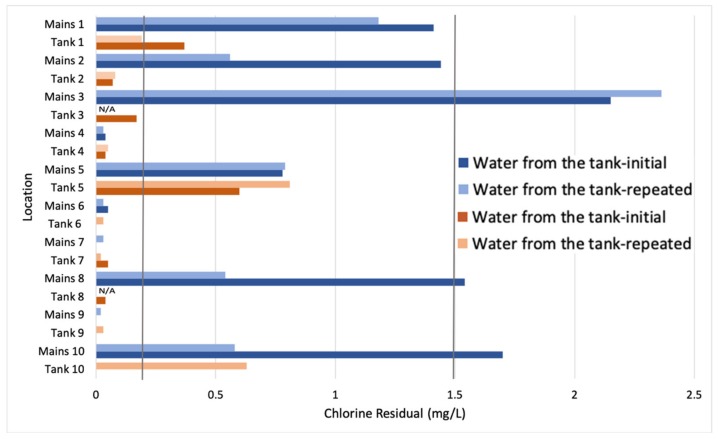
Changes in chlorine residual in the region of the ZMG, supplied by Lake Chapala/SIAPA in July 2018. Measurements were taken at the same time in each location from taps supplied from the water mains (labeled as “Mains”) and the storage tanks (labeled as “Tank”). Mexican drinking water standards require chlorine residual to be between 0.2 and 1.5 mg/L; this range is demarcated by the vertical lines in bold.

Chlorine residuals of the water supplied directly from the SIAPA infrastructure varied greatly throughout the ZMG (Figure 3). For houses that participated in the microbial testing, only the initial chlorine residual value is shown. In five locations, chlorine residuals exceeded the local standard of 1.5 mg/L. In 26 locations, chlorine residuals were below the minimum local standard of 0.2 mg/L. Of the 51 homes sampled, only 18 homes had chlorine residuals that met the local permissible range of 0.2 to 1.5 mg/L. Figure 4 displays houses with chlorine residual results that met the standard in blue, houses that were below the standard (0.2 mg/L) in yellow, and above the standard (1.5 mg/L) in red. Figure 5 shows the results of the chlorine residual testing from the 10 sites chosen for microbial analysis. Chlorine residual measurements were highly variable between sample sites and between days. Given the small sample size, a statistical analysis was not conducted.

### 3.3. Microbial Analysis

The results of the microbial analysis are presented in Table 3. No changes in results occurred between the 24 and 28 h analysis times. No *E. coli* was detected. However, at the exterior taps of two homes (representing water from the SIAPA infrastructure), total coliform counts exceeded the local standards of no detectable organisms in 100 mL. Samples taken from taps supplied by the water tanks at five of the 10 homes showed contamination. Three of the five homes which exhibited higher than acceptable total coliform counts had rooftop tank models without antibacterial coating. Two of the homes which exhibited higher than acceptable total coliform counts in the water drawn from the tanks, but no more than 1.1 MPN in the water drawn from the mains, reported a low frequency of cleaning the water tanks. One home reported cleaning the tank once 12 months prior to testing and a relatively constant water supply, but it appears that the antibacterial coating in the tank was insufficient to prevent contamination.

## 4. Discussion

**Figure 6:**
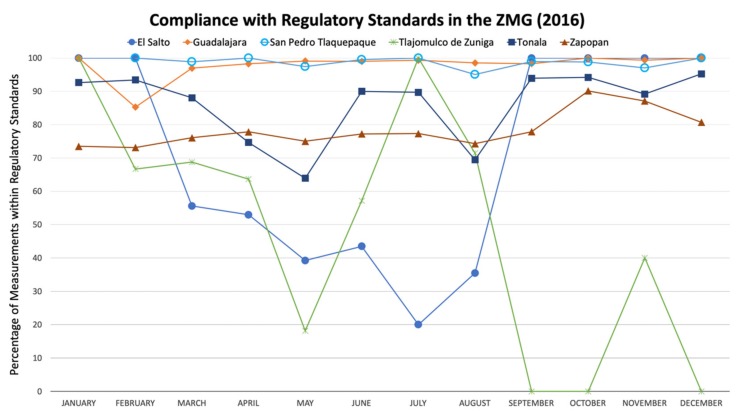
Monthly variation in water quality in 2016 throughout the ZMG, assessed by Mexican regulatory standards according to NOM-127-SSA1-1994 [23].

The results of this pilot study suggest that there may be serious issues concerning the water quality in the ZMG that merit further investigation. Many households reported fluctuations in service from daily cuts to annual losses of service. Participants stated that these events were often associated with unpleasant colors and odors. The decline in water quality reported to occur yearly in April and May is consistent with the marked decline in water quality that occurred during the end of the dry season in 2016, as reported by COFEPRIS, Mexico’s Federal Commission for the Protection against Sanitary Risk (Figure 6) [23]. On average, the dry season begins in October and ends as the rainy season begins in May [24]. Although such aesthetic problems are not always harmful, they can be related to contamination. The high chlorine residual in some areas was reported as alarming to residents who complained that their water smelled overwhelmingly of chlorine. We observed a broad range in chlorine residual (both high and low), as well as fluctuations from one day to the next, at the same time and location. These results suggest that disinfection practices in the system may need improvement to supply water that is both palatable and safe.

The effect of the region’s intermittent water supply may have an impact on health because of the household storage of water in rooftop tanks. Half of the tanks tested positive for total coliform bacteria. Although the tanks tested negative for *E. coli*, Colilert™ does not test for all pathogenic serotypes of *E. coli*. The World Health Organization describes coliform bacteria as a good test for the assessment of disinfection practices but not necessarily an indicator of health risk [25]. *E. coli* is considered the best indicator of fecal contamination. However, both of these bacterial tests are limited in their ability to reveal the presence of other microbes such as viruses and protozoa. Our study indicated that water storage systems, and thus the intermittent water supply that causes these devices to be necessary, may be a contributing factor to the exposure to microbial contaminants. More comprehensive bacterial testing, including speciation of coliform bacteria, would shed light on the extent of the microbial contamination in rooftop tanks in the ZMG. Furthermore, the survey showed that the public had low confidence in the quality of water supplied by SIAPA. The lack of perceived access to clean drinking water may have serious health implications if the lack of perceived access to clean water encourages the consumption of sugary drinks [26]. Such behaviors are associated with serious adverse health outcomes, such as diabetes, obesity, cardiovascular disease, and tooth decay [27,28].

As a pilot study, there were limitations in our ability to assess the overall microbial quality and public perception of water in the ZMG. The difficulty in randomizing participation in the study and the limited sample size mean that the heterogeneity in water quality and perception in the ZMG were not fully captured. During the survey, some respondents indicated that they did not use the water for cooking but were observed using the water to boil food. Furthermore, only adults were interviewed. In at least one home, children drank the water without a parent’s permission, suggesting that the number of people who are exposed to the water may be higher. It can be difficult to maintain proper conditions during the transportation of samples from the field in warm, rainy climates and improvements in field testing conditions could improve results. Finally, the incubator’s design could be improved to limit samples’ exposure to light. Limited access to equipment is a common issue in testing water quality in low-resourced settings. While temperatures were closely monitored throughout transport and incubation, future work to address bacterial contamination may be improved if access to laboratory incubation or testing is made available for a follow-up study. A broader incubating temperature range has been shown to not significantly impact the performance of the Colilert™ test to detect *E. coli*, but has been shown to impact detection of total coliforms [20]. Regardless, these results demonstrate the real need to further evaluate the water quality that is supplied to over five million people.

## 5. Conclusions

In this limited study, drinking water in homes in Guadalajara was found to be contaminated with coliform bacteria, and/or had a chlorine residual outside of regulatory norms. Future studies are needed to understand the complicated issues concerning water quality in the ZMG. A longer study would help evaluate the effects of the intermittent water supply and the risk of contamination. A study should be done to determine an appropriate cleaning schedule for the tanks to prevent re-contamination during storage. In addition to further studies needed to characterize the impact storing water in roof-top tanks or cisterns has on water quality, environmental scientists will need to work together with city planners and community members to not only provide clean drinking water but to improve the infrastructure and build the public’s appreciation of water.

## Figures and Tables

**Table 1:** Survey results on public perception of drinking water.

Description	Yes	No	% (Yes) Usage of Tap Water
Drinking	4 ^a^	57	7
Washing fruits & vegetables	58 ^b^	3	95
Cooking	12 ^c^	49	20
**Observed water aesthetic**			
Unpleasant odors	28	33	46
Unpleasant colors	36	25	59

^a^ One home drank the water after additional filtration; ^b^ Includes households which take extra precautionary steps, such as additional filtration or adding disinfectant; ^c^ Includes households which take extra precautionary steps, such as additional filtration or boiling.

**Table 2:** Survey results on intermittent water supply device.

Description	n ^a^	%	Description	n ^a^	%
Storage device			Last tank cleaning		
Rooftop tank	27	44	<1 month	7	17
Cistern	2	3	1–6 months	11	22
Both	32	52	6–12 months	12	24
Tank type			12–24 months	8	16
Concrete/plastic	44	86	25–60 months	2	4
Asbestos	7	14	>5 years	12	24
Cistern type			Last cistern cleaning		
Concrete/plastic	22	65	<1 month	5	15
Asbestos	11	32	1–6 months	8	26
Frequency of tank cleaning			6–12 months	10	29
>1 year	23	46	12–24 months	4	12
1–3 years	6	12	25–60 months	1	3
3–5 years	3	6	>5 years	8	24
>10 years	19	37			
Frequency of cistern cleaning					
>1 year	16	47			
1–3 years	3	9			
3–5 years	0	0			
>10 years	15	44			

^a^ Number of answers.

**Table 3:** Colilert-18™ Results. Most Probable Number (MPN) is calculated using 10, 10mL test tube analysis except where noted. “Mains” indicates samples drawn from taps supplied by the water mains and “Tanks” indicates samples drawn from taps supplied by rooftop storage tanks.

Mains Chlorine (mg/L)	Tank Chlorine (mg/L)	Mains MPN	Tank MPN	Tank with Antibacterial Coating
1.41	0.37	<1.1	9.2 ^a^	No
1.44	0.07	1.1	<1.1	Yes
2.15	0.17	<1.1	<1.1	No
0.04	0.04	>23	>23	No
0.78	0.6	<1.1 ^b^	<1.1	No
0.05	0	1.1 ^c^	<1.1	Yes
0	0.05	1.1	5.1	Yes
1.54	0.04	<1.1	<1.1	Yes
0	0	>23	>23	Yes
1.7	0	<1.1	3.6	No

^a^ MPN is calculated from 8 samples; ^b^ One test tube only contained 5mL; ^c^ MPN is calculated from 9 samples. Chlorine results presented are those collected on the first day.

## References

[1] United Nations International Children’s Emergency Fund, Organisation Mondiale de la Santé (2015). Progress on Drinking-Water and Sanitation: 2015 Update and MDG Assessment.

[2] Bain R., Gundry S., Wright J.A., Yang H., Pedley S., Bartram J. (2012). Accounting for Water Quality in Monitoring Access to Safe Drinking-Water as Part of the Millennium Development Goals: Lessons from Five Countries. Bull. World Health Organ..

[3] Onda K., Lobuglio J., Bartram J. (2013). Global access to safe water: Accounting for water quality and the resulting impact on MDG progress. World Health Popul..

[4] Bain R., Cronk R., Wright J., Yang H., Slaymaker T., Bartram J. (2014). Fecal Contamination of Drinking-Water in Low- and Middle-Income Countries: A Systematic Review and Meta-Analysis. PLoS Med..

[5] Kumpel E., Nelson K.L. (2013). Comparing microbial water quality in an intermittent and continuous piped water supply. Water Res..

[6] Ayoub G.M., Malaeb L. (2006). Impact of intermittent water supply on water quality in Lebanon. Int. J. Environ. Pollut..

[7] Tokajian S., Hashwa F. (2003). Water quality problems associated with intermittent water supply. Water Sci. Technol..

[8] Erickson J.J., Smith C.D., Goodridge A., Nelson K.L. (2017). Water quality effects of intermittent water supply in Arraijan, Panama. Water Res..

[9] Ercumen A., Gruber J.S., Colford J.M. (2014). Water Distribution System Deficiencies and Gastrointestinal Illness: A Systematic Review and Meta-Analysis. Environ. Health Perspect..

[10] Ercumen A., Arnold B.F., Kumpel E., Burt Z., Ray I., Nelson K., Colford J.M. (2015). Upgrading a Piped Water Supply from Intermittent to Continuous Delivery and Association with Waterborne Illness: A Matched Cohort Study in Urban India. PLoS Med..

[11] Abo-Shehada M.N., Hindyia M., Saiah A. (2004). Prevalence of Cryptosporidium parvum in private drinking water cisterns in Bani-Kenanah district, northern Jordan. Int. J. Environ. Health Res..

[12] Cifuentes E., Solano M., Santos R. (2002). Diarrheal diseases in children from a water reclamation site in Mexico City. Environ. Health Perspect..

[13] Ramírez G. Área Metropolitana de Guadalajara|Gobierno del Estado de Jalisco. https://www.jalisco.gob.mx/es/jalisco/guadalajara.

[14] Insitituto Nacional de Estadística y Geografía Censo de Población y Vivienda 2010. http://www.beta.inegi.org.mx/proyectos/ccpv/2010/.

[15] Von Bertrab E. (2003). Guadalajara’s water crisis and the fate of Lake Chapala: A reflection of poor water management in Mexico. Environ. Urban..

[16] Sistema Intermunicipal de los Servicios de Agua Potable y Alcantarillado Informe de Actividades y Resultados 2017. http://www.siapa.gob.mx/sites/default/files/doctrans/informe_de_actividades_-_anual_2017.pdf.

[17] Rice E.W., Baird R.B., Eaton A.D. (2017). Standard Methods for the Examination of Water and Wastewater.

[18] Bartram J., Ballance R. (1996). Water Quality Monitoring: A Practical Guide to the Design and Implementation of Freshwater Quality Studies and Monitoring Programs.

[19] Diario Oficial De La Federación Modificacion a la Norma Oficial Mexicana NOM-127-SSA1-1994, Salud Ambiental. Agua Para Uso y Consumo Humano. Límites Permisibles de Calidad y Tratamientos a que Debe Someterse el Agua Para su Potabilización. http://www.salud.gob.mx/unidades/cdi/nom/m127ssa14.html.

[20] Matthews R.L., Tung R. (2014). Broader incubation temperature tolerances for microbial drinking water testing with enzyme substrate tests. J. Water Health.

[21] Bain R., Bartram J., Elliott M., Matthews R., McMahan L., Tung R., Chuang P., Gundry S. (2012). A Summary Catalogue of Microbial Drinking Water Tests for Low and Medium Resource Settings. Int. J. Environ. Res. Public Health.

[22] Stauber C., Miller C., Cantrell B., Kroell K. (2014). Evaluation of the compartment bag test for the detection of *Escherichia coli* in water. J. Microbiol. Methods.

[23] COFEPRIS Calidad del Agua de Uso y Consumo Humano. https://datos.gob.mx/busca/dataset/calidad-del-agua-de-uso-y-consumo-humano.

[24] Fonseca-Hernández M., Tereshchenko I., Mayor Y.G., Figueroa-Montaño A., Cuesta-Santos O., Monzón C. (2018). Atmospheric Pollution by PM_10_ and O_3_ in the Guadalajara Metropolitan Area, Mexico. Atmosphere.

[25] Galal-Gorchev H., Ozolins G., Bonnefoy X. (1993). Revision of the WHO guidelines for drinking water quality. Annali Dell’istituto Superiore Sanita.

[26] Onufrak S.J., Park S., Sharkey J.R., Sherry B. (2014). The Relationship of Perceptions of Tap Water Safety with Intake of Sugar Sweetened Beverages and Plain Water among U.S. Adults. Public Health Nutr..

[27] Malik V.S., Popkin B.M., Bray G.A., Després J.-P., Hu F.B. (2010). Sugar-sweetened beverages, obesity, type 2 diabetes mellitus, and cardiovascular disease risk. Circulation.

[28] Wiener R.C., Shen C., Findley P.A., Sambamoorthi U., Tan X. (2017). The association between diabetes mellitus, sugar-sweetened beverages, and tooth loss in adults: Evidence from 18 states. J. Am. Dent. Assoc..

